# Visualization of Cerebrospinal Fluid Outflow and Egress along the Nerve Roots of the Lumbar Spine

**DOI:** 10.3390/bioengineering11070708

**Published:** 2024-07-12

**Authors:** Diana Vucevic, Vadim Malis, Won C. Bae, Hideki Ota, Koichi Oshio, Marin A. McDonald, Mitsue Miyazaki

**Affiliations:** 1 Department of Radiology, University of California San Diego, La Jolla, CA 92093, USA; dvucevic@health.ucsd.edu (D.V.); v2malis@health.ucsd.edu (V.M.); wbae@health.ucsd.edu (W.C.B.); mamcdonald@health.ucsd.edu (M.A.M.); 2 Department of Radiology, VA San Diego Healthcare System, San Diego, CA 92161, USA; 3 Department of Radiology, Tohoku University, Sendai 980-8576, Miyagi, Japan; hideki.ota.d6@tohoku.ac.jp; 4 Department of Radiology, Juntendo University, Tokyo 113-8421, Japan; koichi.oshio@me.com

**Keywords:** spinal cerebrospinal fluid, nerve root, non-contrast spin-labeling MRI, time–spatiallabeling inversion pulse (Time-SLIP), subarachnoid space

## Abstract

Intrinsic cerebrospinal fluid (CSF) dynamics in the brain have been extensively studied, particularly the egress sites of tagged intrinsic CSF in the meninges. Although spinal CSF recirculates within the central nervous system (CNS), we hypothesized that CSF outflows from the lumbar spinal canal. We aimed to visualize and semi-quantify the outflow using non-contrast MRI techniques. We utilized a 3 Tesla clinical MRI with a 16-channel spine coil, employing time–spatial labeling inversion (Time-SLIP) with tag-on and tag-off acquisitions, T2-weighted coronal 2D fluid-attenuated inversion recovery (FLAIR) and T2-weighted coronal 3D centric *k_y_-k_z_* single-shot FSE (cSSFSE). Images were acquired using time–spatial labeling inversion pulse (Time-SLIP) with tag-on and tag-off acquisitions with varying TI periods. Ten healthy volunteers with no known spinal diseases participated. Variations in tagged CSF outflow were observed across different thoracolumbar nerve root segments in all participants. We quantified CSF outflow at all lumbar levels and the psoas region. There was no significant difference among the ROIs for signal intensity. The tagged CSF outflow from the spinal canal is small but demonstrates egress to surrounding tissues. This finding may pave the way for exploring intrathecal drug delivery, understanding of CSF-related pathologies and its potential as a biomarker for peripheral neuropathy and radiculopathy.

## 1. Background

While MRI has been extensively used to study CSF movement in the brain using phase contrast and spin labeling [1,2,3,4,5,6], the study of spinal CSF flow remains largely under-investigated. CSF is a clear fluid that circulates around the brain and spinal cord, providing a cushioning effect and possibly facilitating the exchange of nutrients and waste products between the central nervous system (CNS) and the bloodstream [7]. The majority of CSF is produced in the lateral ventricles of the brain by specialized ependymal cells and flows through interventricular foramina to the third ventricle, into the fourth ventricle via the cerebral aqueduct and finally to the subarachnoid spaces via the foramen of Magendie and Luschka [8,9]. In the cranial subarachnoid space, CSF circulates rostrally to the villous sites of absorption or caudally to the spinal subarachnoid space via the foramen magnum, which is located at the base of the skull [10]. The fluid then moves down the spinal canal, filling the subarachnoid space and bathing the spinal cord [11].

The potential clearance pathways of CSF remain an area of active scientific debate. In the brain, the majority of CSF reabsorption occurs through the arachnoid villi and granulations in the cranial venous sinuses [12]. Recent studies have identified additional egress points in small animals using injectable tracers such as gadolinium-based contrast agents (GBCAs) including potential egress via the diploic space, a perineural pathway along the olfactory nerves at the cribriform plate as well as via meningeal lymphatics in the subpial space [13]. A recent study by Malis et al. [14] utilized a novel CSF spin-labeling perfusion-like technique to investigate the age-related decline in intrinsic CSF outflow metrics at the superior sagittal sinus (SSS) through the meninges and uncovered significant findings regarding the dynamics of fluid outflow in this area. The study identified intrinsic CSF outflow from the perivascular space of vessels via parasagittal dura (PSD) to the SSS with potential influences from various factors such as the geometry of the SSS, the compliance of the meninges and the pressure gradients within the CSF. As proof of concept, Miyazaki et al. [15] extended this line of inquiry by demonstrating the ability of physical exercise to alter intrinsic CSF outflow metrics and potentially recruit alternate CSF egress pathways at the PSD. These reports provided important insights not only into the mechanisms of fluid outflow in the CNS but also into the validity of non-invasive, non-contrast MR techniques in the study of CSF throughout the remainder of the neuroaxis.

Recent in vitro and animal model research has shed new light on the potential mechanisms of CSF reabsorption in the spine, highlighting the potential roles of arachnoid granulations, perivascular spaces and lymphatic vessels in maintaining spinal cord health and facilitating the clearance of waste products from the CNS [16,17,18,19]. However, CSF flow dynamics and clearance in the spine have been, to date, far less studied in human subjects. Nevertheless, the use of phase contrast (PC) and time–spatial labeling inversion pulse (Time-SLIP) imaging has revealed important insights into bulk flow in the intracranial and intraspinal canal [4,6] and recirculating Lagrangian flow observed in the spinal canal [13]. In terms of CSF egress pathways, it is widely acknowledged that CSF exits at the spinal nerve root level [19], a finding corroborated by imaging techniques including myelography and CT angiography [20]. Nevertheless, debate remains as to whether spinal CSF egress occurs via venous routes, lymphatic routes or both, with any future insights having potential impacts on intrathecal drug delivery and pathologic disease states such as root cause of CSF leak, idiopathic intracranial hypertension and arachnoiditis.

The purpose of this study is to investigate whether intrinsic CSF outflow from the spinal canal can be detected using our proposed non-contrast methods [14,15]. Our study is guided by the following two primary hypotheses: (1) Non-contrast MRI techniques, specifically time–spatial labeling inversion pulse (Time-SLIP) with tag-on and tag-off subtraction, can effectively visualize and semi-quantify CSF outflow at the spinal nerve root level; and (2) this imaging method can identify potential CSF/interstitial fluid (ISF) egress pathways from the lumbar spinal canal into the adjacent paraspinal musculature that are not being recirculated within the CNS.

## 2. Methods

### 2.1. Experimental Design

The study was approved by our Institutional Review Board of University of California San Diego. Written informed consent was obtained from all participants in accordance with ethical standards. This was a pilot investigation into the presence of spinal CSF outflow and did not employ randomization or a priori sample size calculation. All experiments were performed in accordance with the relevant guidelines and regulations.

### 2.2. MR Protocol

All MR imaging data were acquired on a clinical 3 Tesla MR imager (Vantage Galan 3T, Canon Medical Systems Corp., Otawara, Tochigi, Japan), equipped with 16-channel spine coil. A total of 10 healthy subjects (6 males and 4 females; age range 19–48 years; mean ± standard deviation age, 31 ± 10 years) were recruited for the study. T2-weighted coronal 2D fluid-attenuated inversion recovery (FLAIR) and T2-weighted coronal 3D fan-shaped centric *k_y_-k_z_* radar-like trajectory single-shot fast spin echo (3D cSSFSE) was employed to localize the thoracic and lumbar regions of the spinal canal [14,15,21]. T2-weighted axial imaging was also performed to locate Time-SLIP. Images were acquired using a tag-on and tag-off alternate acquisition for the inversion time (TI) of 50, 150, 300, 500, 750, 1000, 1500 and 2000 ms [13,14]. To avoid blood contamination, a long TE_eff_ of 300 ms was used in the Time-SLIP experiments, as arterial blood T2 values are reported to be ~175 ms at 3T [22], depending on the hematocrit, oxygenation and temperature [23,24]. All MR imaging parameters are listed in Table 1.

Investigation of the driving force of intracranial CSF movement showed significant movement associated with respiration compared to the cardiac cycle [5]. Additionally, the supine and prone position altered CSF movement, with less compliance observed in intraspinal CSF [4]. At the lumbar spine level, CSF flow is relatively slow compared to the cervical and thoracic spine levels [13]. Therefore, we believe that respiration or cardiac cycle pulsation does not significantly alter CSF movement at the lumbar spine level; hence, our imaging was performed without gating. Our imaging technique using a zigzag centric *k_y_-k_z_* trajectory is shown to be motion robust in coronary MRA [21]. It is important to note that extreme body movements, such as tumbling upside down in gymnastics, could potentially alter CSF outflow. However, all subjects in our study maintained a regular lifestyle without such activities.

**Figure 1:**
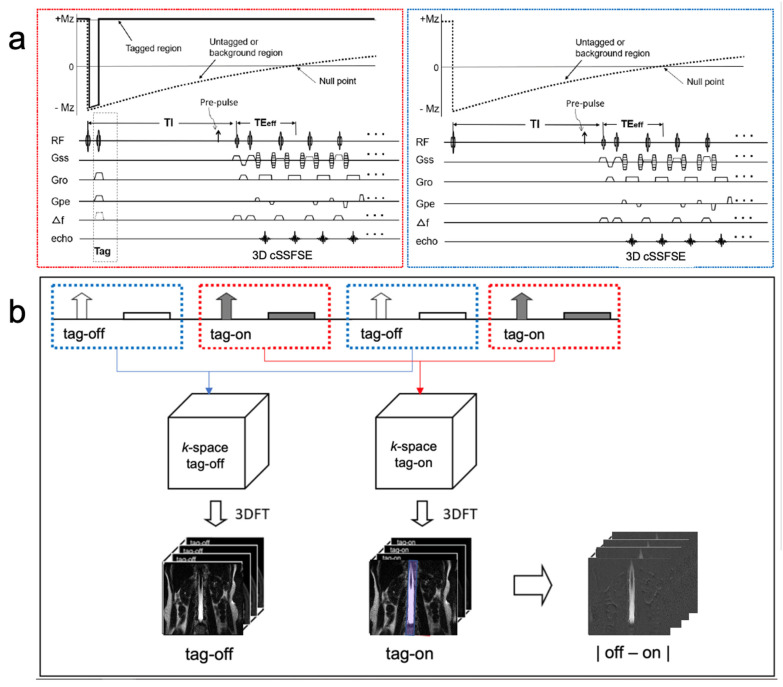
Sequence diagram of 3D Time-SLIP sequence and (**b**) reconstruction and subtraction. An alternate tag-on and tag-off acquisition is shown. (**a**) The tag-on acquisition (red dotted box) consists of a non-selective IR pulse immediately followed by a selective IR pulse. The tag-off acquisition (blue dotted box) consists of only non-selective IR pulses. (**b**) Each tag-on and tag-off acquisition was Fourier transformed to an image, and then followed by the subtraction. The tagged region is +Mz magnetization throughout the TI period and elsewhere, or the untagged region experiences an exponential return in both tag-on and tag-off acquisition. Therefore, the same exponential signals will be canceled out by the subtraction. The 2 cm tag pulse in purple applies to the center of the lumbar spinal cord. (Modified with permission Ref. [14].)

Four-dimensional time-resolved or three-dimensional spin-labeling MRI with time involves the alternating acquisition of “tag-on” and “tag-off” images at each TI. Figure 1a shows the sequence diagram of tag-on and tag-off acquisition. The tag-on acquisition consists of two essential components: a non-selective inversion recovery (non-sel-IR) pulse and a spatial-selective inversion recovery (sel-IR) pulse. First, the non-sel-IR pulse inverts the magnetization across the entire field of view (FOV), transitioning it from a positive longitudinal magnetization state (+Mz) to a negative state (−Mz). Following this, the sel-IR pulse is applied immediately, spatially selectively inverting only the magnetized tissue and fluid within a designated tagged region. This process effectively restores the longitudinal magnetization within the tagged area to +Mz, while magnetization in other regions continues to follow an exponential T1 relaxation recovery curve. The tag-off acquisition, in contrast, involves the application of only the non-sel-IR pulse. Both the tag-on and tag-off acquisitions are collected alternately, as shown in Figure 1b. The critical aspect of this technique is the subsequent subtraction analysis of these acquisitions. By subtracting the tag-off images from the tag-on images, the technique highlights the flow-out signals generated by the tag pulse. Importantly, both tag-on and tag-off acquisitions incorporate the non-sel IR pulse ensuring the background signals with exponential T1 relaxation characteristics remain consistent in both images. Consequently, these background signals effectively cancel each other out during subtraction, leaving only the tagged signal, which represents the flow of interest moving away from the tag or Time-SLIP pulse. This differential tagging allows for the isolation of CSF signals by subtracting the tag-off images from the tag-on images, resulting in tagged CSF movement of the tagged region to be depicted clearly after subtraction.

The tag was positioned in the sagittal plane, with a 2 cm width. The region of interest (ROI) was on the nerve root of the lumbar spine to investigate intrinsic CSF outflow. The ROIs were selected on a TI of 500 ms Time-SLIP image because of the superior image contrast of the 500 ms image between the CSF and the background signals. An area of 1.264 cm^2^ was used for every ROI to minimize variation in the signal intensity measurements. Relative to the spinal canal, ROI 1, 3, 5 and 7 were proximal with the corresponding distal ROIs labeled as 2, 4, 6 and 8, some of which included the psoas muscle.

The quantification of CSF outflow was performed using the signal increase ratio (SIR) of the tagged spinal cord and CSF region MRI in the spinal canal. To determine quantitative measures of CSF outflow, we calculated SIR using the following formula:

SIR = | SI_Tag-on_ − SI_Tag-off_ |/| (SI_Tag-off at TImax_) |

At each TI, SI_Tag-on_ and SI _Tag-off_ are the signal intensity of tag-on and tag-off images, respectively. We divided by the maximum TI of a tag-off image, and the process was repeated, leading to a signal increase over time for each voxel. The average values of the ROI were subsequently utilized to conduct a least-squares fitting analysis using a TI-dependent gamma variate function [25]. The fitting procedure yielded estimations for metrics peak height (PH) and time-to-peak (TTP). The detailed methodology of data analysis and post-processing was explained in our previous article [14]. In addition, the visualization of spinal CSF outflow was attempted using the SIR with the subtraction of tag-on and tag-off images of the tagged spinal cord and CSF region MRI in the spinal canal. The subtraction process involves the pixel-wise subtraction of tag-off images from tag-on images. This method cancels out static background signals, highlighting the dynamic flow of CSF as it moves away from the tagged region, thereby enhancing the visualization of CSF outflow.

### 2.3. Statistical Analysis

Normality was assessed using the Shapiro–Wilk test, which is suitable for small sample sizes. Upon confirming the normal distribution of the data, we proceeded with parametric statistical tests. Specifically, we employed Analysis of Variance (ANOVA) followed by Tukey’s post hoc pairwise comparisons with 5% significance level. ANOVA was chosen because it allowed us to compare the means of multiple groups (different ROIs) simultaneously, providing an overall test of significance. Tukey’s post hoc test was used to control for Type I errors when making multiple comparisons between groups. Additionally, Pearson correlation analysis was conducted to examine the relationships between signal intensity ratios (SIRs) across different ROIs. This analysis helps in understanding the consistency and potential physiological relevance of our measurements across various segments. Statistical testing was performed using GraphPad Prism 9 (GraphPad software, San Diego, CA, USA).

## 3. Results

**Figure 2:**
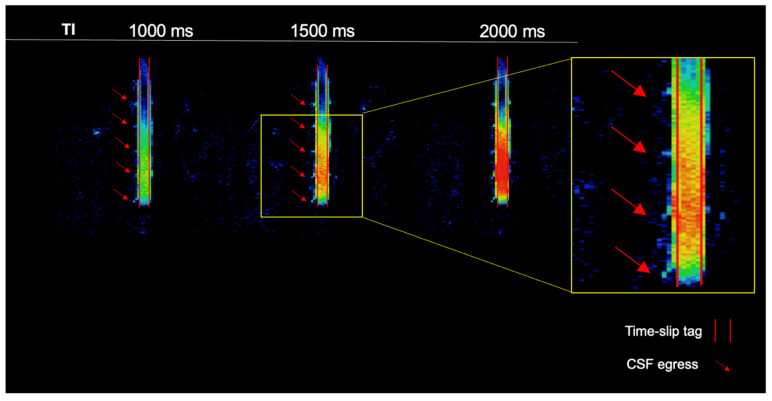
Simple subtraction colormap of a healthy volunteer. The oblique sagittal tag with a 2 cm width is shown in the red parallel lines. At 1000 ms and 1500 ms, we observe pronounced CSF egress of the thoracolumbar region. CSF outflow is shown with red arrows.

**Figure 3:**
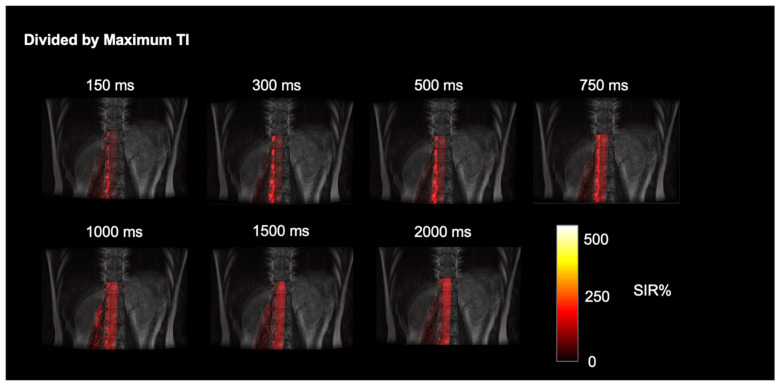
Time-resolved dynamic CSF outflow images fused with FLAIR images. SIR images at each TI, of subtracted images of tag-on and tag-off divided by maximum TI. The tagged CSF outflow from the nerve roots and egress over the psoas muscle is shown.

The simple subtracted images of tag-on and tag-off images of a volunteer, as shown in Figure 2, reveal pronounced CSF egress at 1000 ms and 1500 ms between TI of 150 and 2000 ms from the tag region in the thoracolumbar nerve roots. The time-resolved CSF outflow, detailed in Figure 3, further illustrates the movement of CSF from the spinal cord, traveling out of the nerve roots and into the surrounding psoas muscle. Figure 3 shows the tag-on and tag-off subtracted images divided by the maximum TI. The SIR images at each TI show tagged signals even farther from the nerve root going to the psoas muscle. A video of Figure 3 is shown in the Supplementary Materials (Figure S1).

**Figure 4:**
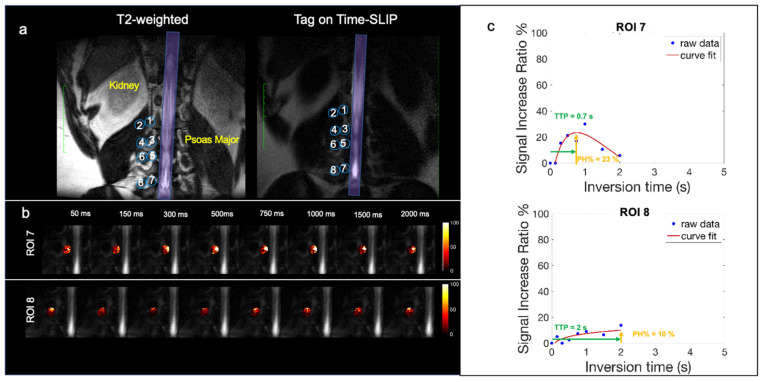
Healthy volunteer CSF outflow. (**a**) Segmentation of 8 ROIs along the thoracolumbar nerve roots with the corresponding T2 image for segmentation reference. (**b**) Fusion images of signal increase ratio (SIR) over tag-off image from TI 50 ms to 2000 ms for ROI 7 and ROI 8. (**c**) SIR graph of measured data (blue dots) and curve fitting (red).

Figure 4a displays a coronal T2-weighted image with the corresponding tag-on Time-SLIP image, which incorporates eight ROIs placed at the lumbar region on the right side spanning the L1-L4 nerve roots of a healthy volunteer without lower back pain. Figure 4b displays ROI 7 and ROI 8 dynamic flow signals at each TI. We observe CSF flow into ROI 7 and then travel into the distal ROI 8. Figure 4c shows the perfusion curves. ROI 7, close to the spinal canal, has a PH of 23% and TTP 0.7s. ROI 8 shows a smaller plateau curve, which is consistent with all the distal ROIs, indicating that the flow is distributing into the psoas muscle slowly. ROI 8 distal to the spinal canal has a PH of 10% and TTP of 2 s. The mean PH and TTP of the ROIs are recorded in Table 2.

Statistical testing of PH and TTP were performed on ANOVA followed by Tukey pairwise comparison of all ROIs. No significant difference between the ROIs for PH and TTP was found. This highlights the consistency and uniformity of CSF outflow across various spinal segments in healthy volunteers. Such findings reinforce the robustness of our technique and provide a reliable baseline for future studies involving pathological conditions. The Pearson correlation analysis of PH across the ROIs reveals strong positive correlations. ROI 1 and ROI 7 have a correlation coefficient of 0.85, ROI 4 and ROI 7 have a correlation coefficient of 0.87, and ROI 6 and ROI 7 have a correlation coefficient of 0.88. Most correlation coefficients are close to 1, indicating that an increase in PH value in one ROI is generally associated with an increase in PH values in other ROIs. The correlation between levels in the spine and medial and distal ROI correlation could suggest points of recirculation, and/or multiple pathways from the medial nerve root to distal ROIs, egressing to different lumbar levels. Lastly, we observed that ROI 7 (L4 medial) had the highest PH signal out of the rest of the ROIs.

**Figure 5:**
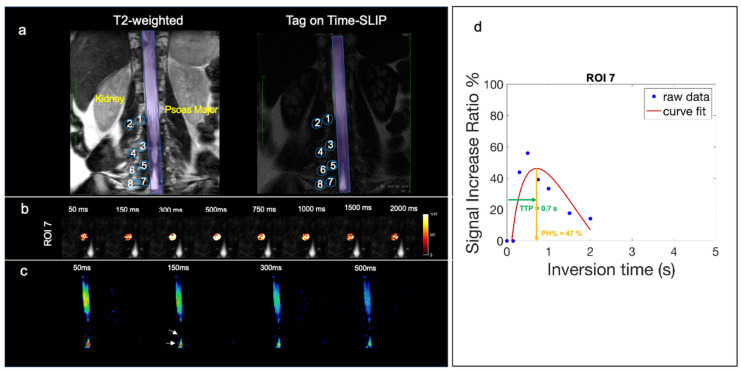
Volunteer with back pain in lower lumbar region. (**a**) Segmentation of 8 ROIs along the thoracolumbar nerve root with the corresponding T2 image for segmentation reference. (**b**) Fusion images of signal increase ratio (SIR) over tag-off image for ROI 7. (**c**) Simple subtracted images of tag-on and tag-off images. The arrow indicates the location of observed CSF outflow from nerve roots. (**d**) SIR graph of measured data (blue dots) and curve fitting (red). High PH% of 47% and TTP of 0.7 s.

Out of the 10 volunteers scanned, 1 had indicated generalized lower back pain. The volunteer with generalized lower back pain had an elevated CSF signal in the L4 ROI 7 spinal nerve root, which contributed to the highest SD. Figure 5a shows the coronal T2-weighted image with the corresponding Time-SLIP image in a subject with back pain localizing to the paraspinal region overlying ROI 7 (L4). Figure 5b demonstrates CSF outflow in this location at the TI of 300 ms. The subtraction image in Figure 5c shows the CSF outflow in the lower lumbar region, exiting the nerve root at ROI 7 (L4). The PH was 47%, as shown in Figure 5d of the signal curve, much higher than the volunteers with no back pain. Though the scope of this study is limited to healthy volunteers, these results are encouraging for future studies that focus on a patient cohort of spinal disease. Reproducibility graphs for two volunteers were scanned in Supplementary Figure S2. Tests and retests were performed more than 3 months apart. In volunteer 1, the test and retest were examined before and after gallstone surgery, resulting in greater differences in measurements. In addition, the metrics may vary by various conditions such as physical activity, sleep effect, etc.

## 4. Discussion

Building on our established work with tagged CSF in the meninges [14,15], we have applied this technique to the spinal canal. In this study, we used a TE of 300 ms compared to a TE of 30 ms to eliminate blood contamination. Additionally, we placed a sagittal tag over the entire spinal canal. Our results provide proof of concept for the utilization of non-contrast MRI to identify and visualize CSF outflow at the level of the spinal nerve roots in the lumbar region in all participants. Previously reported methods like PC and 2D Time-SLIP flow imaging have their own limitations. The PC technique allows qualitative measures of flow at a relatively fast bulk flow like at an aqueduct [1,2,3]; however, PC lacks the spatial resolution and sensitivity to visualize slower, more subtle CSF movements, especially at the spinal nerve root level. The 2D Time-SLIP method allows the depiction of the CSF bulk flow of intracranial sites including aqueduct, foramen of Monro and prepontine cistern and intraspinal CSF bulk flow within the spinal canal as up–down movement [4]. However, its ability to observe the tagged CSF in the spinal canal as it moves out via the nerve root to the psoas muscle has not been reported. We have also been successful in visually identifying the intrinsic CSF outflow and observing direct egress into the surrounding paraspinal soft tissues/musculature, as evidenced by CSF signal ROI 7 and ROI 8 leading into the psoas muscle. In addition, using non-invasive, non-contrast techniques, we demonstrate both proximal to distal and cranial to caudal flow gradients. The signal intensities across the ROIs reveal a gradient in CSF flow, with a noticeable decrease from proximal to distal segments, suggesting that tagged CSF from the spinal canal into the surrounding muscle and tissue via nerve roots. The rhythmic pattern of CSF outflow, particularly at 1000 ms and 1500 ms marks, underscores the pulsatile nature of spinal fluid movement. This observation may potentially reflect recently characterized closed recirculation CSF flow by W. Coenen et al. [13] who investigated CSF bulk flow patterns in the spinal canal in six human subjects utilizing phase-contrast techniques. The authors identified three distinct patterns of CSF flow—pulsatile, oscillatory and transitional—each with its own characteristics and velocity profile. The patterns varied significantly between individuals and were influenced by factors such as the geometry of the spinal canal with associated subject-specific alterations in CSF distribution. The authors demonstrated that thoracic spinal nerve T11 and T12 are areas of relatively increased CSF recirculation and, potentially as a result, lower CSF outflow based on metrics obtained using our noninvasive technique, which is supported by the recirculation within the lumbar region [13].

In terms of SIR metrics, we calculated only PH and TTP. The reason for this is that the tagged CSF outflow does not exhibit typical blood perfusion characteristics, which often display rapid signals with up-and-down patterns. Instead, it demonstrates a rapid signal-up followed by a prolonged signal-down phase, unlike blood perfusion. This pattern, characterized by a high PH value, is illustrated in Figure 5d, showcasing the subject with lower back pain.

Another recent study by Gutiérrez-Montes et al. [26] investigated the movement of CSF in the spinal canal during normal respiration in a small number of subjects. The findings showed that the CSF moves in a cyclic manner that corresponds to the breathing pattern, with the largest stroke volume found in the lower thoracic and upper lumbar spine and decreasing towards the foramen magnum and the sacrum. In this study, the largest net volume variation, and therefore spinal CSF flow, was found in the upper lumbar segment, consistent with our own findings.

We found an elevated CSF signal in the spinal nerve root from the subject with back pain and an elevated psoas CSF signal from the subject with arthritis. Although these very early observations will need to be reproduced, it is tantalizing to suggest that this finding may reflect CSF/ISF accumulation at the site of localized spinal pain. Recent research [27,28] has suggested that the CSF itself may play a role in the regulation of immunity and inflammation in part by providing transport for a variety of cytokines and both T cells and natural killer cells. As such, one hypothesis for this observation is that the upregulation of CSF flow and volume may be a potential reactive or even homeostatic mechanism with which to trigger inflammatory states and/or clear out waste products in the spine, akin to its postulated role in the brain.

This study has several limitations. To further investigate the role of CSF outflow including the phenomenon of CSF passing through or perfusing in degenerative and inflammatory spine disease, more subjects should be recruited with dedicated neurological examinations to establish correlations with the potential dermatomal distribution of pain states as well as pain onset and duration. We observed perfusion-like curves within 2 s in areas closer to the CNS, whereas distal areas have a prolonged signal which may be caused by the slow movement of tagged CSF in the areas. A longer TI may be required to observe the signal return to the baseline. Our current technique is limited to studying CSF, which cannot differentiate between CSF and/or ISF. Furthermore, the present proof-of-concept study was limited to right-sided lumbar segmentation; future work will repeat investigations on the contralateral side as well as extend the investigation to cervical and thoracic segments.

Our work is the first to validate the use of non-invasive, non-contrast MR techniques to quantify and visualize CSF outflow in the spine as a surrogate for CSF/ISF egress. Our results demonstrate CSF outflow along the lumbar nerve roots as the first direct visualization of dispersed CSF egress from the nerve root sheaths. Further studies with larger and more diverse populations are essential to substantiate these findings and to explore their potential implications for the design and delivery of intrathecal drugs, the identification of CSF flow abnormalities related to CSF leaks and other causes of CSF hypotension, as well as their utility as biomarkers for peripheral neuropathy and radiculopathy.

## Figures and Tables

**Table 1:** MR acquisition parameters.

Parameter	Units	T2 FLAIR	3D SSFSE	3D Spin Labeling
Field of view	[mm]	350 × 250 × 48	350 × 250 × 52	350 × 250 × 64
Image matrix		288 × 256 × 16	320 × 256 × 52	368 × 368 × 16
No. of slices		16	26	16
Slice thickness	[mm]	3	2	4
Acquired resolution: (PE) × (RO) × (SE)	[mm]	1.21 × 0.97 × 3.0	1.09 × 0.97 × 2.0	0.95 × 0.68 × 4.0
Interpolated resolution: (PE) × (RO) × (SE)	[mm]	N/A	0.54 × 0.48 × 1.0	0.47 × 0.34 × 2.0
Repetition time	[ms]	10,000	4000	~10,000
Effective echo time	[ms]	92	117	300
Echo train spacing	[ms]	11.5	6.5	5
Flip/refocusing angles	[degrees]	90/170	90/140	90/160
Averages		1	1	1
Parallel reduction factor		2.4	3	3
Acquisition time		1 min 50 s	2 min	1 min 34 s (for each TI)

**Table 2:** CSF outflow metrics in healthy volunteers.

ROI	PH %	TTP [s]
1 (L1 medial)	17.65 ± 7.7	1.03 ± 0.64
2 (L1 distal)	14.46 ± 12.06	1.55 ± 0.68
3 (L2 medial)	10.1 ± 2.03	0.98 ± 0.62
4 (L2 distal)	17.79 ± 8.29	1.67 ± 0.74
5 (L3 medial)	13.88 ± 6.25	1.09 ± 0.83
6 (L3 distal)	17.92 ± 7.57	1.64 ± 0.51
7 (L4 medial)	20.41 ± 12.62	0.94 ± 0.71
8 (L4 distal)	15.52 ± 11.04	1.69 ± 0.59

Average ± SD.

## Data Availability

All data required to achieve the conclusions in the paper are presented in the paper. None of the material has been published or is under consideration for publication elsewhere. Additional data can be shared on request from qualified investigators for the purposes of replicating procedures and results and for other noncommercial research purposes within the limits of the participants’ consent. Correspondence and material requests should be addressed to mimiyazaki@health.ucsd.edu.

## Supplementary Materials

The following supporting information can be downloaded at: https://www.mdpi.com/article/10.3390/bioengineering11070708/s1, Figure S1: Time-resolved video of CSF egress; Figure S2: Reproducibility graphs.
