# Supplementary material for: Visualization of Cerebrospinal Fluid Outflow and Egress along the Nerve Roots of the Lumbar Spine
Source: Bioengineering (Basel). 2024 Jul 12;11(7):708. doi: 10.3390/bioengineering11070708 (PMC11273714; doi:10.3390/bioengineering11070708)

Supplementary Materials

Figure S1. Time-resolved video of CSF egress.

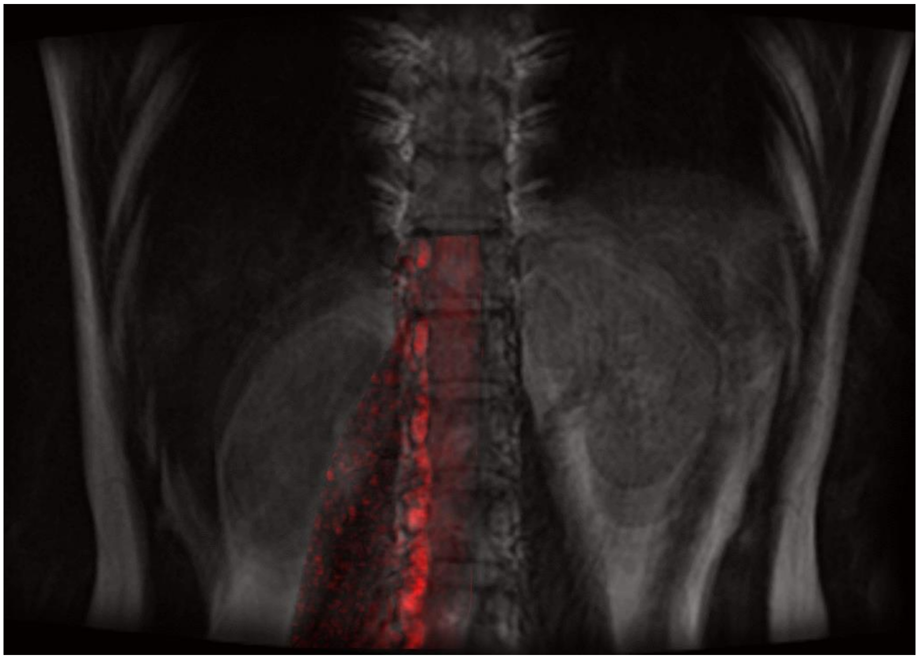

Figure S2. Reproducibility graphs

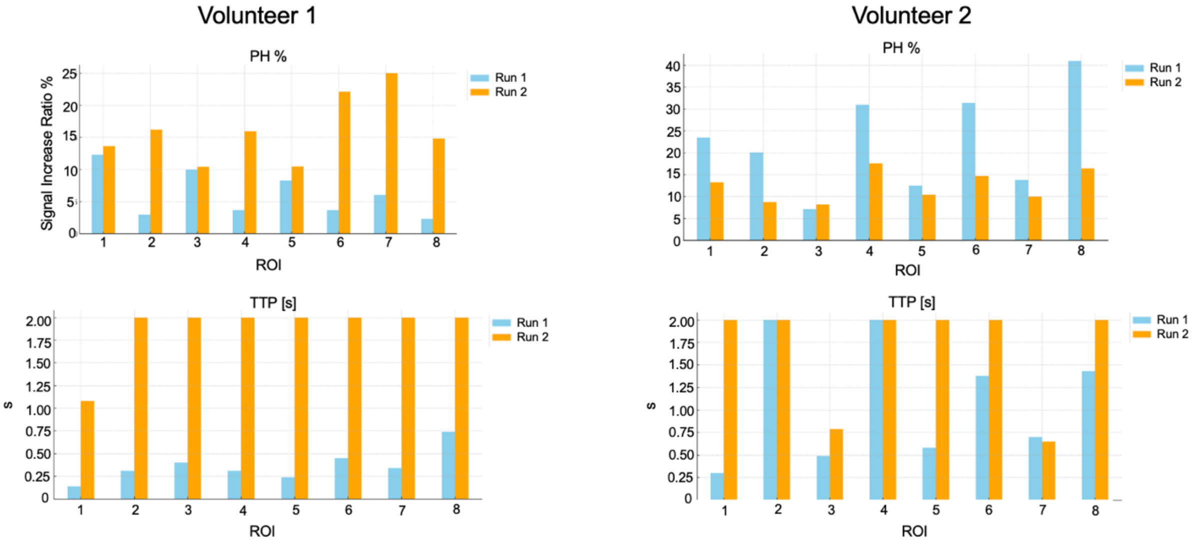

Supplement: Supplementary file 1 [file bioengineering-11-00708-s001.zip › bioengineering-3068146-supplementary.pdf]
